# Lineage-specific energy and carbon metabolism of sponge symbionts and contributions to the host carbon pool

**DOI:** 10.1038/s41396-021-01165-9

**Published:** 2021-12-07

**Authors:** I. Burgsdorf, S. Sizikov, V. Squatrito, M. Britstein, B. M. Slaby, C. Cerrano, K. M. Handley, L. Steindler

**Affiliations:** 1 Department of Marine Biology, Leon H. Charney School of Marine Sciences, University of Haifa, Haifa, Israel; 2 GEOMAR Helmholtz Centre for Ocean Research Kiel, RD3 Marine Ecology, RU Marine Symbioses, Kiel, Germany; 3 Department of Life and Environmental Sciences, Polytechnic University of Marche, Ancona, Italy; 4 School of Biological Sciences, The University of Auckland, Auckland, New Zealand

**Keywords:** Metabolism, Functional genomics, Bacterial genetics, Comparative genomics, Water microbiology

## Abstract

Marine sponges host a wide diversity of microorganisms, which have versatile modes of carbon and energy metabolism. In this study we describe the major lithoheterotrophic and autotrophic processes in 21 microbial sponge-associated phyla using novel and existing genomic and transcriptomic datasets. We show that the main microbial carbon fixation pathways in sponges are the Calvin–Benson–Bassham cycle (energized by light in Cyanobacteria, by sulfur compounds in two orders of Gammaproteobacteria, and by a wide range of compounds in filamentous Tectomicrobia), the reductive tricarboxylic acid cycle (used by Nitrospirota), and the 3-hydroxypropionate/4-hydroxybutyrate cycle (active in Thaumarchaeota). Further, we observed that some sponge symbionts, in particular Acidobacteria, are capable of assimilating carbon through anaplerotic processes. The lithoheterotrophic lifestyle was widespread and CO oxidation is the main energy source for sponge lithoheterotrophs. We also suggest that the molybdenum-binding subunit of dehydrogenase (encoded by *coxL*) likely evolved to benefit also organoheterotrophs that utilize various organic substrates. Genomic potential does not necessarily inform on actual contribution of autotrophs to light and dark carbon budgets. Radioisotope assays highlight variability in the relative contributions of photo- and chemoautotrophs to the total carbon pool across different sponge species, emphasizing the importance of validating genomic potential with physiology experimentation.

## Introduction

Sponges (phylum Porifera) are ancient cosmopolitan filter feeders [1, 2]. They play an important role in nutrient recycling by transforming dissolved organic matter (DOM) into detrital particulate organic matter, thereby making it available for other invertebrates in nutrient poor environments [3, 4]. Symbiotic microbial communities of 268 different sponge species include more than 60 bacterial and archaeal phyla in total [5], with all sponges hosting symbionts of at least 13 different phyla [6]. Sponge symbionts are often specific to one or a few hosts [6] with the exception of a few cosmopolitan symbiont species that are found in diverse hosts around the globe [6–10]. These sponge-associated symbionts can be categorized based on their nutritional strategies, for instance (photo- and chemo-) autotrophic, organoheterotrophic, and lithoheterotrophic. Photoautotrophic and chemoautotrophic organisms harvest energy from light or inorganic compounds for inorganic carbon fixation. Autotrophically fixed carbon may later be used as the main carbon source by other organoheterotrophic or lithoheterotrophic microorganisms that co-occur in the same sponge. Despite relying on the external carbon supply for biomass, lithoheterotrophic organisms can gain energy from inorganic sources.

Heterotrophic symbionts can contribute up to 87% of the total sponge holobiont DOM assimilation [11], whereas autotrophic photosymbionts can contribute to host growth when exposed to light [12]. Various bacterial and archaeal phyla in sponges in addition to photosymbionts harbor mechanisms associated with autotrophic metabolism. Autotrophic fixation of inorganic carbon (Ci) can occur via six known pathways including the Calvin–Benson–Bassham (CBB) cycle, the reductive tricarboxylic acid (rTCA) cycle, the Wood–Ljungdahl pathway (WL), and the 3-hydroxypropionate/4-hydroxybutyrate (3-HP/4-HB) cycle [13]. The 3-HP/4-HB pathway was previously reported in sponge-associated Thaumarchaeota, and the rTCA in bacterial sponge symbionts affiliated with Nitrospirota, Alphaproteobacteria, and Oligoflexia [14–19].

Additionally, various solo-acting enzymes can be involved in Ci assimilation without *sensu stricto* being part of carbon fixation. For instance, Ci assimilation in anaplerotic reactions was proposed to be abundant among marine planktonic heterotrophs [20–22]. Anaplerotic reactions undertaken by pyruvate (PYC) and phosphoenolpyruvate (PPC) carboxylases often occur at low levels to replace intermediates of the tricarboxylic acid (TCA) cycle. However, enhanced anaplerotic Ci assimilation was reported in marine planktonic lithoheterotrophs that combine organoheterotrophy with the additional use of inorganic electron donors [22]. The malic enzyme (MEZ) was also shown to operate in the carboxylating (anaplerotic) direction in *Mycobacterium tuberculosis* [23, 24], in planktonic stages of *Pseudomonas aeruginosa* PAO1 [25], and in deep-sea Alphaproteobacteria [26]. Carbon fixation capacities through the canonical pathways and carbon assimilation *via* solo-acting enzymes remain under-described within the sponge microbiome, and the contribution of chemoautotrophy to the pool of microbially fixed carbon in sponges has not yet been tested.

Net primary productivity and stable isotope analyses of microbial and host sponge fractions showed that different species of symbiotic Cyanobacteria differ in their ability to assimilate and transfer carbon to the host [27]. The unicellular *Parasynechococcus*-like cyanobacterial species are the most commonly reported in sponges [28, 29]. These include *Candidatus* Synechococcus spongiarum, enriched in 28 sponge species around the globe (including *Theonella swinhoei* from this study) [30], and *Candidatus* Synechococcus feldmannii, the symbiont of *Petrosia ficiformis* [31, 32]. The latter symbiosis is facultative, with *P. ficiformis* growing in light environments with *Ca*. S. feldmannii, and in dark-(cave)-environments without it. The heterotrophic microbial community of *P. ficiformis* is functionally and compositionally independent from the presence of *Ca*. S. feldmannii, being nearly identical in both structure and gene expression in specimens with and without this photosymbiont [32, 33]. This suggests that photosynthetically derived carbon may not be the main carbon source for heterotrophic *P. ficiformis*-associated symbionts.

Here, we characterized the dominant carbon fixation processes and identified the energetic sources used by lithoheterotrophs across different microbial species within sponge symbiotic communities. This was achieved through genomic analysis of 402 symbiotic metagenome-assembled genomes (MAGs) from ten different sponge species sampled from different geographic locations, and 39 metatranscriptomes from the sponge *P. ficiformis*. Further, using radioisotopes, we investigated the contribution of light and dark microbial carbon fixation in two sponge systems (*P. ficiformis* and *T. swinhoei*).

## Materials and methods

### Sponge sampling, and microbial DNA extraction and purification

In total, this study analyzed 402 MAGs obtained from ten sponge species. Of these, 56 MAGs were assembled from four sponge species that were collected as part of this study, the remaining 346 MAGs were derived from other studies (Tables S1,  S2). Three *P. ficiformis* specimens, 277c, 287ce, and 288c (c, cortex; e, endosome), and one specimen each of *T. swinhoei*, *Ircinia variabilis*, and *Aplysina aerophoba* were collected by SCUBA diving (Table 1). The samples were immediately preserved in liquid nitrogen for further processing. DNA from *P. ficiformis* was extracted using a phenol-chloroform method as previously described [34]. Microbial DNA was enriched using New England Biolab’s NEB-Next Microbiome DNA Enrichment Kit according to the manufacturer-recommended protocol. DNA extraction from *T. swinhoei*, *I. variabilis*, and *A. aerophoba* are described in ref. [35] and ref. [15], respectively. Six additional sponge species were collected as part of other studies previously published by different research groups (Table S2). All sponges sampled in this study were collected in compliance with permits from the Israel Nature and National Park Protection Authority.

**Table 1:** Sponge samples used in this study.

Sponge species	Use	Number sampled (names)	Collection date	Depth (m)	Geographic location	Latitude and longitude	Bioproject (NCBI)
*Aplysina aerophoba*	MAGs assembly	1 (15)	7/5/2013	5	Slovenia: Gulf of Piran, Adriatic Sea	45°30′35.6″N 13°33′36.0″E	PRJNA712987
*Ircinia variabilis*	MAGs assembly	1 (142)	5/5/2013	7.5	Israel: Achziv Nature Marine Reserve, Mediterranean Sea	33°00′36.0″N 35°02′24.0″E	PRJNA273429
*Petrosia ficiformis*	MAGs assembly	3 (277c, 287ce, and 288c)	6/1/2014	27, 23, and 15	Israel: Achziv Nature Marine Reserve, Mediterranean Sea	33°00′36.0″N 35°02′24.0″E	PRJNA515489
*Theonella swinhoei*	MAGs assembly	1 (SP3)	31/07/2012	25	Israel: Eilat, Gulf of Aqaba, Red Sea	29°30′04.8″N 34°55′04.4″E	PRJNA255756
*Petrosia ficiformis*	Radioactive experiments	3	13/05 and 09/06/2020	22–27.5	Israel: Achziv Nature Marine Reserve, Mediterranean Sea	33°00′36.0″N 35°02′24.0″E	NA
*Theonella swinhoei*	Radioactive experiments	5	2000	25–32	Israel: Eilat, Gulf of Aqaba, Red Sea	29°30′06.6″N 34°55′02.6″E	NA

### Shotgun sequencing, assembly, and binning

Preparation of metagenomic shotgun sequencing KAPA Hyper DNA libraries, sequencing, read trimming, and de novo assemblies for the three *P. ficiformis* specimens were performed as previously described [36]. 50 genomes were binned using manual methods including usage of differential coverage information derived from three *P. ficiformis* specimens (10.6084/m9.figshare.14601321.v1). Taxonomic affiliation of assembled scaffolds from *P. ficiformis*, binning of final MAGs, and relative abundance calculations are described in Supplementary File S1. Out of these 50 MAGs, 48 are novel and 2 were recently published (Tables S2, S3). In addition, eight novel MAGs were assembled from available metagenomes of *T. swinhoei*, *I. variabilis*, and *A. aerophoba* (Tables S2,  S3) [15, 30].

### MAG annotation and completeness estimation

Open Reading Frames were identified using Prodigal v2.6.3 with the metagenome options [37]. Protein sequences were queried against the Clusters of Orthologous Groups (COGs) database (version 2014) as previously described (Supplementary File S2, also available at 10.6084/m9.figshare.14601351.v1) [36]. The amino acid sequences were also searched against the KEGG orthology (KO) database using standalone KofamKOALA 1.3.0 (Supplementary File S3, also available at 10.6084/m9.figshare.14601360.v1) [38]. Selected enzymes were annotated using previously published Hidden Markov models (HMM) [39] with individual score thresholds (Table S4) using hmmsearch [40]. Phylogenomic tree construction and taxonomic annotation was done using PhyloPhlAn2 [41] (https://bitbucket.org/nsegata/phylophlan/wiki/phylophlan2), RAxML [42] as previously described [43], and GTDB-Tk v1.3 with release r95 [44]. Trees were visualized using iTol [45]. Completeness and contamination rates of all final MAGs were estimated with checkM version 1.0.7 [46] using lineage_wf.

### Annotation of transcriptomic data

Metatranscriptomes were previously obtained from 39 *P. ficiformis* specimens sampled in Ligurian Sea, Italy (as described in [32]), and quantified by Salmon software [47]. Translated sequences of the assembled and filtered bacterial metatranscriptomes were assigned to the proteins of MAGs assembled from Israeli *P. ficiformis* specimen 277c using blastp 2.2.30+ (*E*-value threshold = 1E − 10, identity = 55%). Taxonomic annotation of transcripts was determined based on the best hits (highest bit score and lowest *E-*value). Transcripts with the same function and MAG affiliation were merged prior to analyses. Expression of certain functions in specific organisms (MAGs) was additionally confirmed using mapping of the genes against metatranscriptome reads with bbmap tool v 37.62 [48] (minimal identity = 0.70, kmer size = 13) from the BBtools package (https://jgi.doe.gov/data-and-tools/bbtools/), (≥5 reads as a threshold). Functional annotation of the metatranscriptomes of *P. ficiformis* against the COG database was done as described previously [36] and search against the KEGG database was done via the GhostKOALA website using the genus_prokaryotes database (August 2020) [49]. Data were analyzed and visualized using the R packages dplyr, tidyr (http://tidyr.tidyverse.org), ggplot2 [50], ggpubr (https://CRAN.R-project.org/package=ggpubr), plotly [51], reshape2 [52], and superheat [53]. Spearman rank correlation was performed to analyse the relations between *coxL* and *pckA* expressions within the specific Acidobacteria symbiont Acido_2. Final figures were graphically edited using Inkscape (http://inkscape.org). A schematic representation of the bioinformatic analyses is available in Fig. S1. The code used in this study can be found on Github (https://github.com/burgsdorf).

### Carbon fixation measurements in *Theonella swinhoei* and *Petrosia ficiformis* with H^14^CO_3_^−^

Sponge samples for carbon fixation experiments were collected by SCUBA diving, and samples were maintained inside seawater-containing zip-lock bags in a cooler for transport to the laboratory. Five specimens of *T. swinhoei* were collected in Eilat, Red Sea, and three specimens of *P. ficiformis* were collected from the Achziv nature marine reserve in the Mediterranean Sea (Table 1). Photosynthetic (light) and chemosynthetic (dark) fixed carbon measurements were performed on the day of sampling, except for one *P. ficiformis* specimen that was held in a closed aquaria system at University of Haifa, for 47 days prior to the experiment. Aquaria conditions were 12/12 h light/dark regime (15 µmol photons m^–2^ s^−1^) at 22 °C. Carbon fixation is the result of the microbial activity, thus cylinders cut from the same sponge specimen were considered as biological replicates. We are aware that sponge activity may affect the transfer of compounds within its tissue and that the stress caused by cutting a cylinder could affect the results. Therefore, the effect of cylinders on results was tested (see below). The incubation time was determined using one specimen of *T. swinhoei* in the following five steps: In step one, nine sponge cylinders (cylinders, ~0.79 cm^2^ surface area and 1.5 cm depth) were cut perpendicular to the sponge surface, to contain both the Cyanobacteria-containing external layer (with *Ca*. S. spongiarum), as well as internal sponge parts.

In step two, the cylinders, after being cut out of the sponge, were placed in a container of autoclaved seawater and 1 µl of NaH^14^CO_3_ (ARC, 150922, 1 mCi/1 ml) tracer for each 10 ml medium and exposed to 50 µmol photons m^−2^ s^−1^ (light intensity corresponding to that measured in situ when the sponge specimens were collected). Temperature (ca. 20 °C) was maintained by keeping the container in a temperature-controlled water bath at a temperature that reflected that measured at the site of collection. One additional cylinder was treated with formalin (final concentration, 2%) in a separate container and served as a kill control. NaH^14^CO_3_ in the medium was measured every 30 min, by sampling 0.1 ml of seawater from each beaker and transferring to a scintillation vial containing 3 ml of scintillation fluid (Opti-fluor, high flashpoint LSC cocktail, Packard Bioscience). Every 10 min, the water inside the beakers was stirred manually.

In step three, at three-time intervals (30, 70, and 130 min from the beginning of the incubation), three cylinders were removed from the container, and the three consecutive sections (each 2-mm thick) were cut. The first of these sections contained the Cyanobacteria. Each cylinder section was left for 3 min on a paper towel to remove excess water.

In step four, these sections were weighed (for later normalization of data by sponge weight) and transferred each to a separate scintillation vial containing 0.5 ml N,N-dimethylformamide (Sigma) to release labeled, fixed carbon from the tissue to the liquid. Then the samples were acidified with 45 µl 20% HCl (Sigma) to release labeled and unlabeled non-fixed carbon. Sponge tissue was then disintegrated manually with a plastic homogenizer and the vials were left uncovered for 48 h in the chemical hood to allow labeled and unlabeled CO_2_ gas to be completely released. Fixed carbon (e.g., sugars) are not affected by the acidification and remain in the sample.

In step five, after the release of non-fixed carbon from the samples, 0.1 ml liquid was transferred to a new vial containing 3 ml scintillation fluid. Using a liquid scintillation counter (Tri-Carb 1500, Lumitron, Packard Bioscience) discharges per min (DPM) was measured. Based on the obtained results (Fig. S2), we selected 2 h as the incubation time for all subsequent experiments.

In the next experiment, we tested whether results obtained from sponge cylinders were representative of measurements taken when the physiology of the entire sponge was evaluated. For this purpose, two *T. swinhoei* sponge specimens were incubated in glass containers (ca. 2000 ml per container) at the same temperature and NaH^14^CO_3_ conditions as described above for the experiments with sponge cylinders. The experiment was repeated twice (total of four specimens). Each time, one specimen was exposed to a light intensity of 50 µmol photons m^−2^ s^−1^ and the other to darkness. After 2 h of incubation, 7–10 cylinders were cut from each sponge specimen, and 6–7 consecutive 2-mm sections were cut from each cylinder. These sections were processed as described for the experiment above. The amount of fixed carbon in each section was determined for both light and dark exposures in incubations of complete sponge specimens. Results from cylinders and complete sponge (comparing the shared 2-h time point) were similar, and thus experiments on the sponge species *P. ficiformis* were only performed on sponge cylinders, as described below.

*P. ficiformis* is white when it grows in dark underwater caves and does not harbor Cyanobacteria. The presence of Cyanobacteria (*Ca*. S. feldmannii) in the cortex (external sponge layer) cells is indicated by a pink color conferred by the photosynthetic accessory pigments present in the symbiotic Cyanobacteria. Within each *P. ficiformis* specimen, the sponge parts exposed to light will harbor Cyanobacteria and appear pink, while those in the shade will appear white. The following experiment was performed twice, each time on a single *P. ficiformis* specimen (in total, two different sponge specimens) to measure light and dark carbon fixation. Sixteen cylinders in total were cut out of each *P. ficiformis* specimen, of which eight were pink (cortex with *Ca*. S. feldmannii) and eight were white (cortex without *Ca*. S. feldmannii). Of these 16 cylinders, 4 (2 pink and 2 white) were treated with formalin prior to incubation and served as kill controls (they were incubated in separate containers, in light and darkness for both pink and white kill controls). The other 12 cylinders were incubated in light and darkness (3 pink cylinders in light, 3 pink cylinders in darkness, 3 white cylinders in light and 3 white cylinders in darkness). Light intensity was 125 µmol photons m^−2^ s^−1^, the same as measured in situ next to the sponge at the time of sampling. Cylinders were incubated in labeled seawater at 20 °C for 2 h, as previously described (step two of the experimental description above). After the incubation, cylinders were processed as described in steps three to five above, except that the scintillation fluid was UltimaGold, Perkin–Elmer and the counter used was a Tri-Carb 2810TR (Perkin-Elmer). The amount of fixed carbon (µg) was calculated using average DPM of live replicates minus the DPM measured for the kill control. The total amount of labeled carbon in the medium was measured as specific activity (tDPM). Details about the calculations of fixed carbon are available in the Supplementary File S1 (Section: *Calculations of fixed carbon***)**.

An additional (third) *P. ficiformis* sponge specimen was used for determining whether the low dark fixation measured in the previous experiments relates to the fast turnover of fixed carbon. For this purpose, we incubated eight white (Cyanobacteria-free) cylinders in seawater-containing NaH^14^CO_3_ (as for the experiments above) in darkness only. Four in one container and the other four (kill controls treated with formalin) in a separate container. NaH^14^CO_3_ in the medium of each container was measured every 30 min, by sampling 0.1 ml of seawater from each beaker and transferring to a scintillation vial containing 3 ml of scintillation fluid. After 3 h we crushed the live sponge tissues manually with a plastic homogenizer to release fixed carbon and respired ^14^CO_2_, trapped in the tissue, to the medium. After 5 h we added N,N-dimethylformamide for further release of labeled carbon to the medium. NaH^14^CO_3_ in the medium was measured half an hour after the N,N-dimethylformamide amendment (at time point 5.5 h) and finally 16 h from the beginning of the experiment.

## Results and discussion

Overall, 47 bacterial and 3 archaeal MAGs belonging to 14 phyla were recovered from three *P. ficiformis* specimens, and are estimated to be 62 to 100% complete, with 0 to 5.5% contamination (Table S3). These genomes represent 38–41% of the assembled data and were investigated together with eight additionally assembled MAGs from *T. swinhoei* (SP3), *Ircinia variabilis* (142), and *A. aerophoba* (15) and additional 344 MAGs from previous studies [15, 16, 18, 19, 30, 36, 54–63] (Table S2) to identify all the dominant autotrophic and lithoheterotrophic processes found in sponge symbionts.

### Autotrophy in sponge symbionts

**Fig. 1:**
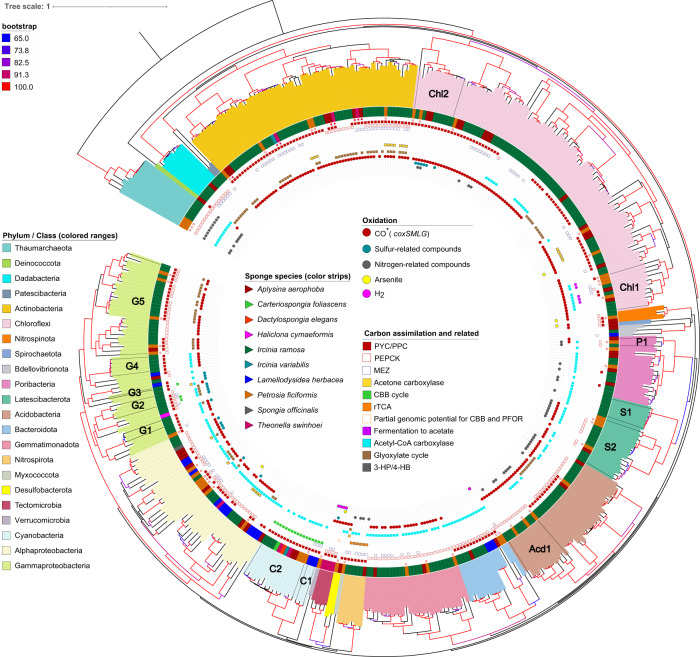
Phylogenomic tree showing the distribution and diversity of carbon assimilation and energy production pathways across microbial symbiont taxonomy and host species. The phylogenomic tree (*N* = 399 MAGs) was constructed based on concatenated universal markers (PhyloPhlAn2). Labels marked with a hollow star are MAGs assembled in this study from the *P. ficiformis* specimen 277c. Labels marked with a colored star are eight MAGs assembled from the *A. aerophoba* specimen 15, *T. swinhoei* specimen SP3 and *I. variabilis* specimen 142. The tree is rooted to the Archaea group. Figure S3 represents an enhanced version (MAGs names are displayed) of this tree. Acd1, class Vicinamibacteria, order *Vicinamibacterales*, family UBA8438. C1, order *Cyanobacteriales*, family *Desertifilaceae*. C2, order *Synechococcales*, family *Cyanobiaceae*. CHL1, class Dehalococcoidia, order UBA3495. CHL2, class Anaerolineae, order SBR1031. G1, order GCA-2729495. G2, order UBA10353, family LS-SOB. G3 (single MAG), order UBA4575. G4, order *Pseudomonadales, Pseudohongiellaceae* family. G5, order *Pseudomonadales*, HTCC2089 family. P1, class and order WGA-4E, unknown family. S1, unknown class. S2, UBA2968 class and order. Poribacteria_ADFK02.1_Kamke_2014, Poribacteria_AQPC01.1_Kamke_2014 and Poribacteria_ASZM01.1_Kamke_2014 were excluded from the phylogenomic tree due to incomplete marker genes set. *CO is not always a target molecule for the *coxSMLG* complex as it was shown here for *Poribacteria*.

**Fig. 2:**
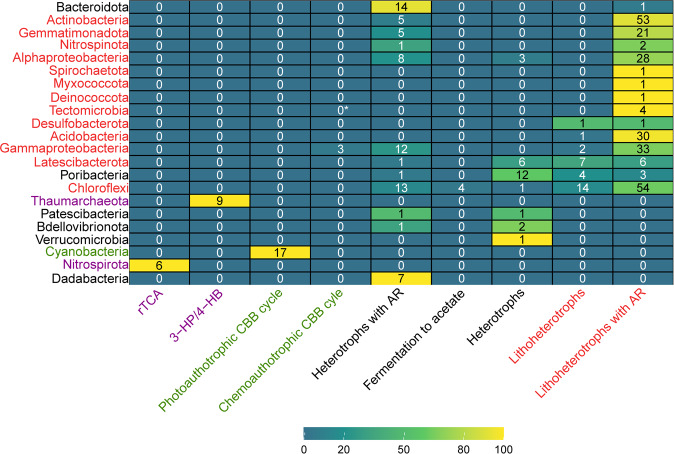
Predicted lifestyle for different taxonomic groups (Phylum/Class) of sponge symbionts. Heat map represents percentage of genomes with predicted lifestyle, text represents number of MAGs. The colors of the MAGs correspond to the most abundant lifestyle: organoheterotrophs (black), lithoheterotrophs (red), autotrophs implementing CBB (green) and other chemoautotrophs (violet). The relevant functions can be found in Table S5. Here, heterotroph means organoheterotroph. AR, anaplerotic reaction. *MAGs of Tectomicrobia (Entotheonella) class possess incomplete genomic potential for utilization of CBB pathways.

We investigated the presence of known prokaryotic carbon fixation mechanisms among 402 sponge-associated MAGs derived from ten sponge species (Figs. 1,  S3, Tables S1, S3, S4). We recovered metabolic capacities of the sponge-associated symbionts (Table S5) and their predicted trophic lifestyles (Fig. 2). Accordingly, we found that the autotrophic pathways 3-HP/4-HB, CBB, and rTCA were mainly restricted to Cyanobacteria, Tectomicrobia, Nitrospirota, and Thaumarchaeota phyla, and two gammaproteobacterial orders.

RuBisCO-related genes are essential for the CBB cycle. These genes were identified in all 16 cyanobacterial and 3 (out of 47) gammaproteobacterial genomes. Gammaproteobacterial MAGs from *P. ficiformis* lacked RuBisCO-related genes, or evidence of any other C-fixation pathways, and thus likely pursued a heterotrophic lifestyle. Yet, within this sponge species, 2 out of 6 gammaproteobacterial MAGs exhibited genomic potential for CO oxidation via carbon monoxide dehydrogenase (CODH), which can serve as an energy source [64]. In *Ircinia ramosa*, Gammaproteobacteria clades G2 (order UBA10353 and family LS-SOB) and G3 (order and family UBA4575) had genes for thiosulfate oxidation (Table S5), which can fuel carbon fixation (Figs. 1,  S3) through RuBisCO (also found in three MAGs within G2 and G3 clades, Supplementary File S1), indicating the potential for chemoautotrophy. Gammaproteobacteria G1, G4 and G5 did not have this pathway, yet encoded for CODH (*coxSML* and *coxG*) (Figs. 1, S3). Taken together, data show that gammaproteobacterial symbionts include two trophic groups: chemoautotrophs and lithoheterotrophs.

The filamentous Entotheonella (phylum Tectomicrobia) found in the sponge *T. swinhoei* [54, 65, 66], were identified as chemoautotrophs based on the presence of a large cohort of CBB related genes [54]. However, we did not detect RuBisCO in these filamentous Tectomicrobia using 4 available genomes (Table S5), which may be due to MAG incompleteness. Energy for carbon fixation in this phylum may be provided by oxidation of multiple inorganic donors, providing metabolic versatility to shifting environmental conditions within the host [67, 68]. Inorganic donors (and mechanisms for oxidation) include CO (CODH), H_2_ (3b group hydrogenase), thiosulfate (Sox complex), and possibly even arsenite (AoxAB) (Table S5). High concentrations of arsenic were reported in *T. swinhoei* compared to the other 15 sponge species [69]. Oxidation of arsenite may have a dual function: energy source [70, 71], as well as detoxification of the highly toxic arsenite to arsenate [72]. Calcium arsenate was in fact observed inside intracellular structures of filamentous Tectomicrobia [65]. Arsenite oxidation is not necessarily limited to filamentous Tectomicrobia, in fact we also found the arsenite oxidase genes (*aoxAB*) in Alphaproteobacteria, Chloroflexi, and Nitrospinota MAGs (Fig. 1, Table S5).

The pyruvate synthase or pyruvate:ferredoxin oxidoreductase (PFOR, EC 1.2.7.1), which is required for the rTCA cycle, can also serve different non-autotrophic functions, such as energy production through fermentation of pyruvate to acetate. For example, sediment Chloroflexi harboring PFOR and acetyl-CoA synthetase [EC 6.2.1.1] were predicted to biosynthesize ATP using this pathway [73]. Here, PFOR was identified in five Chloroflexi MAGs (from *A. aerophoba*, *P. ficiformis*, and *I. ramosa*) that lack carbon fixation pathways and may serve an energy production role. Accordingly, a high abundance of acetyl-CoA synthetase (COG1042) was previously detected in diverse sponge microbial metagenomes [74]. We, therefore, hypothesize that in the studied sponges ATP production involving pyruvate conversion to acetyl-CoA (by PFOR), coupled with acetate formation (by acetyl-CoA synthetase), occurs in five specialized, sponge-associated Chloroflexi (Figs. 1,  S3, Table S5).

### Lithoheterotrophy and metabolism of inorganic compounds in sponge symbionts

We and others have detected genes for oxidation of diverse inorganic compounds, such as CO, nitrite, ammonia, and thiosulfate in the sponge microbial community [18, 75–77]. Here, for the first time to our knowledge, we report the potential for hydrogen oxidation among sponge symbionts, specifically by the Tectomicrobia derived from *T. swinhoei* and in a Bacteroidetes MAG from *P. ficiformis* (Fig. 1, Table S5, Fig. S3). Nitrogen processing by diverse members of the sponge microbiome has been analyzed in several studies (e.g., [75, 77]). It was suggested that ammonia oxidation in sponges is uniquely performed by Thaumarchaeota [77]. Nitrite can be oxidized to nitrate by members of Nitrospirota, Alphaproteobacteria, and Gammaproteobacteria symbionts [18]. We speculate that oxidation of nitrite to nitrate may only be carried out by Nitrospirota, rather than also by Proteobacteria, as previously proposed [18]. We base this speculation on a stricter annotation of the genes involved (*nrxAB*) using both HMM profiles and KEGG annotations (Supplementary File S1).

Orthologues of *amoABC/pmoABC* genes (involved in ammonia and/or methane oxidation [78, 79], Table S4) were here found also in Desulfobacterota (unclassified Deltaproteobacteria based on NCBI taxonomy), specifically in two MAGs deriving from *A. aerophoba* and *P. ficiformis* (Table S5, Supplementary File S1). Based on sequence similarity, we predict that *amoABC/pmoABC* of Desulfobacterota are involved in methane oxidation (Supplementary File S1). Besides methane to methanol oxidation (EC 1.14.18.3), these MAGs also have the potential to further oxidize methanol to formaldehyde (EC:1.1.2.10) (Table S5). Genomic potential for methane to formaldehyde oxidation was previously discovered in sponges, but was not affiliated with members of Desulfobacterota [77, 80]. *amoABC/pmoABC* subunits were shown to be also expressed within the sponge *P. ficiformis* (details provided below).

### CO oxidation in sponge symbionts

CO-oxidizing bacteria are lithoheterotrophs common in sponge microbiomes. Large (CoxL, COG1529) and middle (CoxM, COG1319) subunits of the molybdenum-rich aerobic form of CODH (Mo-CODH) are highly overrepresented in sponge-associated *versus* seawater microbial metagenomes [76]. Mo-CODH has been identified in gamma and alphaproteobacterial sponge symbionts [75, 76] and found to be expressed among phylogenetically diverse symbionts including Actinobacteria, Chloroflexi, and Proteobacteria [81]. Yet the function of CoxL is variable, and its homologues are not solely responsible for CO oxidation. In fact, CoxL was shown to comprise two different forms (I and II), with form II (putative *coxL*) being involved in functions alternative to CO oxidation [64]. To establish the extent to which CO oxidation is abundant in sponge symbionts, determine potential alternative substrates beyond CO, and provide this information at a taxonomic level, we set the following criteria: (i) genomic potential for CO oxidation was based on the presence of 4 subunits (*coxSMLG*) within MAGs, (ii) substrate specificity was based on clustering and reannotation of 2406 translated *coxL* genes against the KO database and on reannotation of transcripts, and (iii) taxonomy of transcripts was defined according to MAG affiliation.

**Fig. 3:**
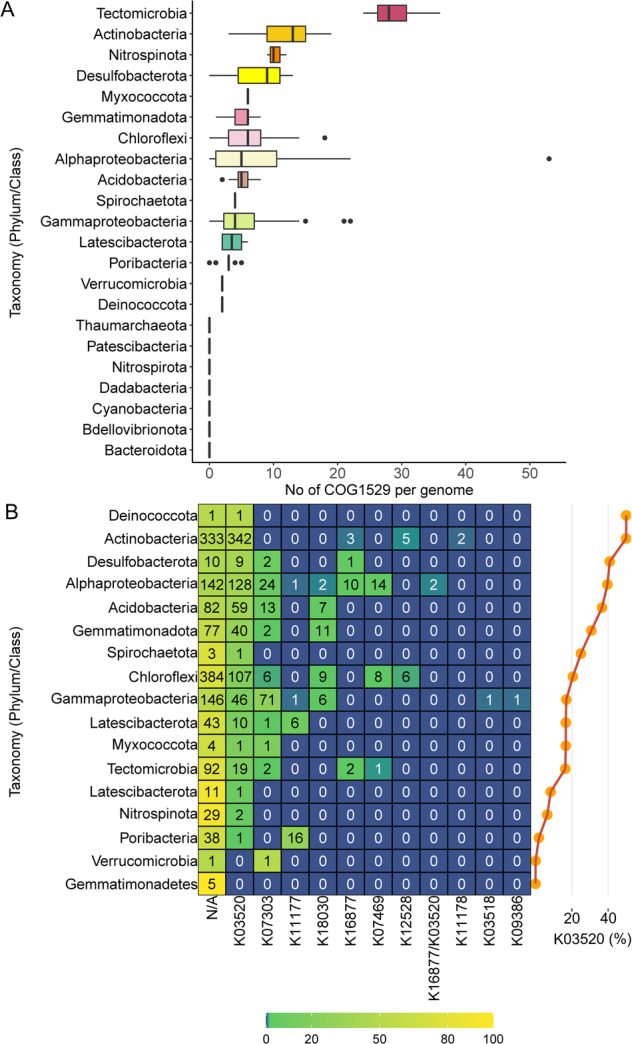
Functional diversity and distribution of COG1529 orthologs across symbiotic bacterial phyla. **A** Number of proteins annotated as COG1529 per genome in different taxonomic groups (Phylum/Class) of sponge symbionts. **B** Functional diversity of the COG1529 orthologous group. Heat map represents percentages of genes with various functions (KEGG annotation) for different taxonomic groups (Phylum/Class) of sponge symbionts. Text represents number of sequences. Percentages of CO-oxidizing *coxL* (K03520) out of total COG1529 are presented on the right. K03520, CO; K07303, isoquinoline; K11177, xanthine; K18030, nicotinate; K16877, 2-furoyl-CoA; K07469, aldehyde; K12528, selenate; K11178, xanthine; K03518, CO; K09386, CO.

**Fig. 4:**
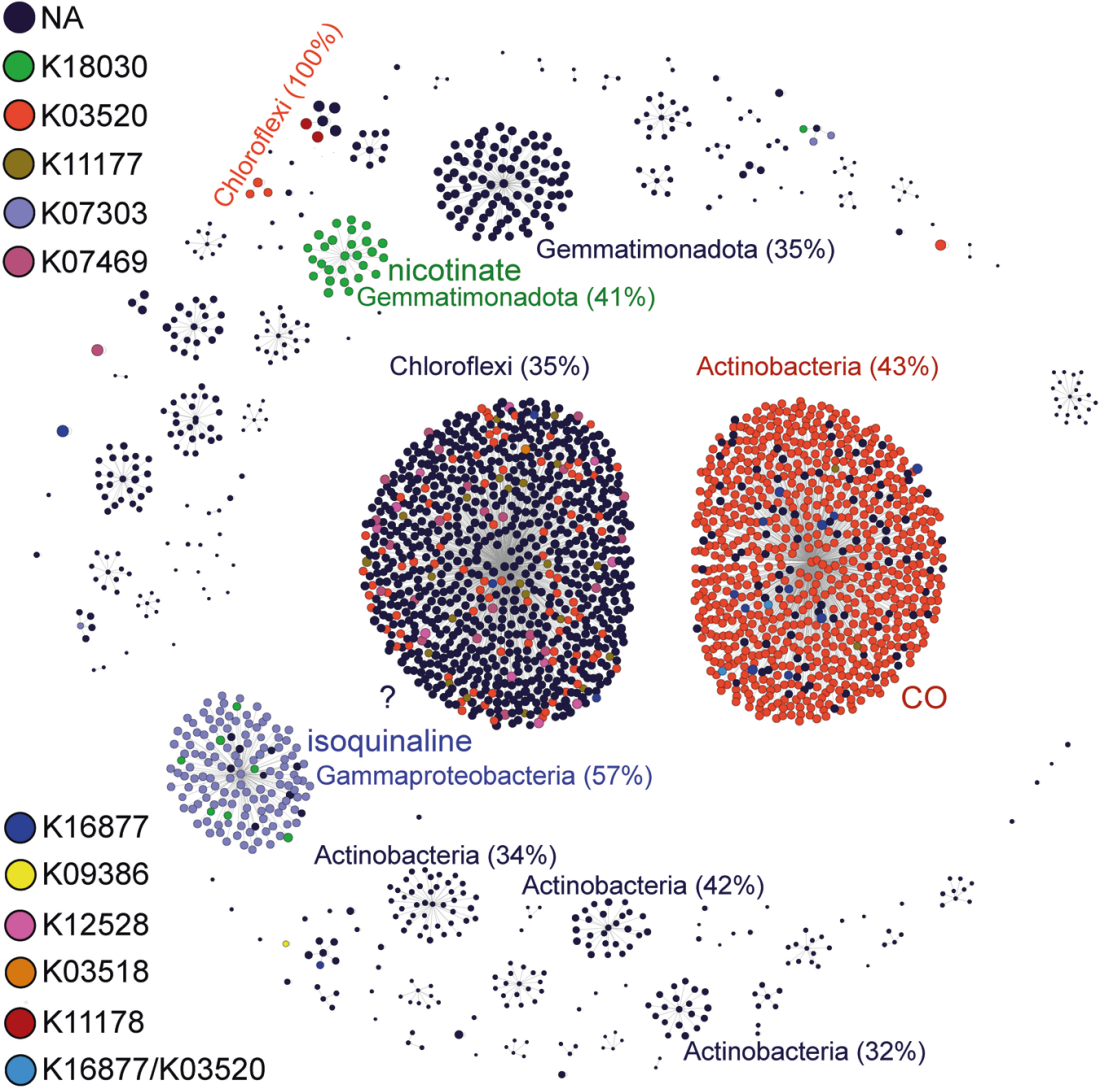
Taxonomic affiliation and hypothesized substrate for CoxL (COG1529) across diversity of sponge-associated MAGs (*N* = 402). Visualization of sequence-based clustering of 2406 proteins annotated as COG1529. Size of the dots is proportional to the length of the protein (in the range of 35–1250 amino acids, average = 682, SD = 207 amino acids). 720 out of 867 sequences forming the largest group (the predominantly black cluster) have unknown function. 674 out of 784 sequences forming the second largest group (the predominantly orange cluster) were annotated as Mo-binding subunit of the CO dehydrogenase (K03520). Percentages represent the most abundant phylum in the cluster. NA not assigned, K18030 nicotinate, K03520 CO, K11177 xanthine, K07303 isoquinoline, K07469 aldehyde, K16877 2-furoyl-CoA, K09386 CO, K12528 selenate, K03518 CO, K11178 xanthine.

The Mo-CODH complex was found in 64% of all analyzed symbionts (Fig. 1, Table S5), suggesting that CO oxidation is the most abundant process related to a lithoheterotrophic lifestyle in sponge symbionts. Overall, more than half of the protein sequences annotated as CoxL COG1529 belonged to Actinobacteria (29%) and Chloroflexi (22%), while Tectomicrobia and Actinobacteria had the highest average number of *coxL* genes (associated COG1529) per genome (Average = 29, SD = 5 and Average = 12, SD = 4, respectively) (Fig. 3A). Among the two largest clusters, one is predicted to function as CO dehydrogenase (the mostly-orange cluster dominated by Actinobacteria, Fig. 4), while the second large cluster could not be linked to any known function (the predominantly black cluster, where Chloroflexi prevail). Additional substrates for CoxL are likely isoquinaline (mostly violet cluster, dominated by Gammaproteobacteria) and nicotinate (green cluster, where Gemmatimonadetes and Chloroflexi prevail). Results therefore suggest that sponge symbionts can gain electrons from CO (lithoheterothrophs) and organic molecules (e.g., isoquinaline and nicotinate; organoheterotrophs) via genes related to a large orthologous group—CoxL COG1529. Nevertheless, the substrate for more than half of the proteins annotated as CoxL COG1529 in sponge symbionts, remains unknown (black dots, Fig. 4; N/A in Fig. 3B).

While we showed here an extensive incidence of CODH within the sponge microbiome, some phyla were found to lack this functional capacity. Specifically, phyla with inherently autotrophic lifestyles (Cyanobacteria, Nitrospirota, and archaeal Thaumarchaeota) (Fig. 2) and phyla specialized in the degradation of polysaccharide residues (Bacteroidota [82, 83], Dadabacteria [84], and Verrucomicrobia [43, 85]), which did not contain CODH (Fig. 3A). An exception are the 2 out of 17 Poribacteria, also characterized as degraders of diverse carbohydrate sources originating in the sponge matrix [62, 77, 86], which harbored CODH (Clade P1 in Fig. 1). However, CoxL in these two Poribacteria might function in the oxidation of xanthin (see below), and they may thus not have a lithoheterotrophic lifestyle. Mo-CODH should be distinguished from Nickel-CODH, which relates to the anaerobic WL pathway. The latter was previously reported in sponge symbionts [15–17, 19], but based on our analysis (combining KEGG, COG, and HMM profiles annotations, Table S4), we conclude that Nickel-CODH, and thus the WL pathway, is absent in the sponge microbiome.

Taken together results indicate that the presence of CoxL COG1529 in sponge symbionts can relate to both CO oxidation (as a part of CODH complex), and thus to a lithoheterotrophic lifestyle, or the oxidation of different organic substrates. Similarly to other symbiotic systems, including the human gut and legumes [64, 87], CO-oxidizing bacteria appear to have an essential role in the sponge holobiont. The potential sources for CO in sponges may include photoproduced CO derived from the ambient seawater [88, 89] and biological hemoprotein degradation via heme oxygenase (HO) activity [64, 87, 90]. Genomic potential for hemoprotein synthesis, transport, and oxidation here found in specific members of the sponge microbiome, and suggested as a potential CO source in sponges, is discussed in Supplementary File S1.

### Gene expression of carbon fixation and energy production pathways: a case study of *P. ficiformis*

To study the activity of key processes related to carbon fixation and energy production from oxidation of inorganic molecules, we conducted a genome-informed metatranscriptomic analysis of the *P. ficiformis-*associated community. We linked 50 MAGs (Table S3) with the previously published assembled metatranscriptome dataset derived from 39 *P. ficiformis* specimens [32]. 35% of transcripts aligned to protein sequences.

Our gene expression results confirm the results derived from the wider MAG analysis described above on the importance of CO oxidation in sponge symbionts, and further corroborate that specific sub-orthologs of COG1529 might provide symbionts with the ability to utilize alternative organic electron donors. The latter may be part of the DOM (or its residues) that is concentrated by the host’s filtration activity [91]. Similar to other sponge species, results show CO-oxidizing bacteria were highly abundant in the sponge *P. ficiformis*, with more than half of the MAGs (64%, *n* = 50) harboring CODH (Fig. 1, Table S5), and with all MAGs affiliated to Actinobacteria (*n* = 13), Acidobacteria (*n* = 4), and Chloroflexi (*n* = 9) harboring CODH. Here, we tested how the widespread genomic potential for CO oxidation relates to its expression across different symbiotic microbial phyla.

**Fig. 5:**
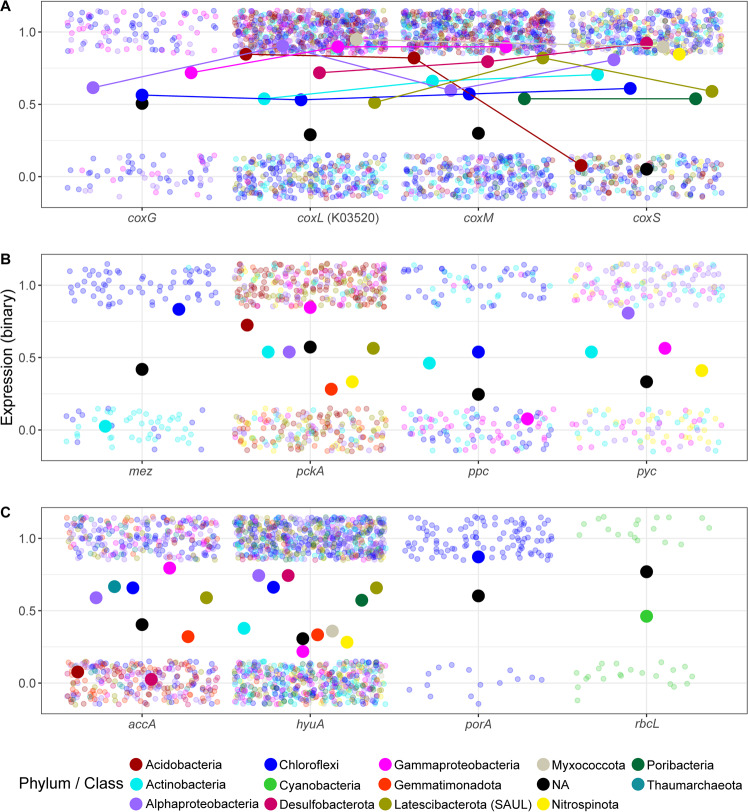
Expression of carbon assimilation and CO oxidation-related functions in the different phyla of *P. ficiformis* symbionts. The analyses are based on cumulative binary (1—expressed, 0—not expressed) expression of transcripts (*N* = 39 transcriptomes). Transcripts with the same function and MAG affiliations are merged. **A** the four subunits of CODH (subunits with the same taxonomy are connected by lines), (**B**) anaplerotic fixation, and (**C**) carbon assimilation genes. Taxonomy of transcripts was assigned if the transcript was linked to the gene of the assembled MAG. Larger dots represent proportion of expression across samples for a certain taxonomy group (Phylum/Class). Transcripts with not assigned (NA) taxonomy (not linked to any assembled MAG) are given as black dots representing mean values. Genes: *mez* malic enzyme, *pckA* phosphoenolpyruvate carboxykinase, *ppc* phosphoenolpyruvate carboxylase, *pyc* pyruvate carboxylase, *accA* subunit of acetyl-CoA carboxylase, *hyuA* subunit of acetone carboxylase, *porA* subunit of pyruvate synthase (PFOR), *rbcL* large subunit of RuBisCO.

We confirmed the expression of CO dehydrogenase (K03520) among eight phyla including Acidobacteria, Actinobacteria, and Chloroflexi (Fig. 5A). Alphaproteobacteria and Chloroflexi expressed all four subunits of CODH, while Acidobacteria, Actinobacteria, Desulfobacterota, and Latescibacterota did not express the *coxG* subunit. The *coxG* gene was also absent from the form 1 (bona fide CO dehydrogenase) Mo-CODH from the chemoautotroph *Alkalilimnicola ehrlichei* MLHE-1 [64], suggesting that the presence of this gene is not crucial for CO oxidation [92, 93]. Interestingly, while the *coxM* and *coxS* subunits of CODH, affiliated to poribacterial MAGs, were expressed, the large CO-oxidizing subunit was absent in the representatives of this phylum. This may be explained by the functional annotation of the CODH complex as xanthine dehydrogenase (EC 1.17.1.4) in Poribacteria (Figs. 3B, 5A, S4), suggesting that this phylum does not oxidize CO in sponges. Functional and taxonomic specialization for certain subgroups of COG1529 was also observed for additional members of the *P. ficiformis* symbiotic microbial community (Fig. S4). For instance, a suborthologous group annotated as a subunit of xanthine dehydrogenase (K13482) was exclusively linked to a single actinobacterial MAG (Actino_4, class *Acidimicrobiia*, order UBA5794, family SZUA-232), and a nicotinate dehydrogenase subunit (K18030) was linked to Gammaproteo_5 (order *Pseudomonadales*, family HTCC2089) (Fig. S4).

It has been suggested that CODH supplies energy for enhanced anaplerotic reactions by PYC in planktonic marine Alphaproteobacteria [21]. Anaplerotic carbon assimilation may contribute differently to the actual biomass accumulation ranging 0.5–1.2% of the total carbon of cells [26] and 10–15% of proteins [94] in different Alphaproteobacteria strains. Due to a high abundance of genes related to anaplerotic reactions (86%) within the fifty *P. ficiformis*-derived MAGs, we next determined the taxa that consistently expressed genes associated with anaplerotic carbon assimilation across multiple *P. ficiformis* specimens. Consistent expression by the same taxon across different specimens implies an enhanced anaplerotic flow, which can result in actual carbon assimilation due to relatively high carbon influx to the TCA cycle, while sporadic expression is more likely to be related to metabolic flexibility with periodical replenishment of TCA intermediates [22]. We observed prevalent expression (≥90% of all samples, *n* = 39) of transcripts showing high similarity to anaplerotic proteins (PYC, MEZ, and phosphoenolpyruvate carboxykinase [PCKA]) belonging to 3 Acidobacteria (order *Vicinamibacterales*), one Alphaproteobacteria (order UBA2966), and one Chloroflexi (order UBA3495). Thus, anaplerotic carbon assimilation in *P. ficiformis* might occur in Acidobacteria, Alphaproteobacteria, and Chloroflexi. When genes were mapped against metatranscriptome reads, only the MAG-specific affiliation of *pckA* from Acidobacteria (MAG Acido_2) was confirmed (Fig. S5). We further observed correlations between expression levels of *coxL* and PCKA transcripts, linked to Acido_2 MAGs, across twelve different *P. ficiformis* samples (Fig. S6). In contrast to PYC, PPC, and MEZ, PCKA utilizes CO_2_ rather than bicarbonate. We hypothesize that in Acido_2 within *P. ficiformis* and, possibly, in closely related *Vicinamibacterales* symbionts of *I. ramosa* (Figs. 1,  S3, Clade Acd1), inorganic carbon assimilation may occur by CoxL supplying CO_2_ to the anaplerotic reaction catalyzed by PCKA.

Genomic potential for CBB was here found in symbionts of the Cyanobacteria and Proteobacterota (Gammaproteobacteria) phyla and was previously reported for Tectomicrobia [54]. Here we investigated the expression of the large subunit of RuBisCO (*rbcL*) in *P. ficiformis*. As expected, all samples that harbored Cyanobacteria (pink phenotype [32], *n* = 12) showed expression of *rbcL*. In addition, we observed expression of a gammaproteobacterial *rbcL* in 30 (out of 39) samples (Fig. 5C). This suggests that the Italian population of *P. ficiformis* (used for the transcriptomics data) is associated with a specific gammaproteobacterial symbiont with CBB activity, providing capability for dark fixation, while the Israeli population of *P. ficiformis* (used for obtaining MAGs) appears to lack this symbiont (Supplementary File S1). A biogeographic effect on the microbial composition of *P. ficiformis* was reported before [33, 35].

Microbial carbon fixation can also occur through the 3-HP/4-HB cycle, which was suggested to be energetically fueled by ammonia oxidation in sponge-associated archaea [77]. Thaumarchaeota MAGs from *P. ficiformis* harbored *amoABC* genes (Fig. 1, Table S5) and expressed *amoC* (Fig. S7A), as well as acetyl-CoA/propionyl-CoA carboxylase, the key gene of the 3-HP/4-HB cycle (Fig. 5C). These findings confirm the involvement of Thaumarchaeota in dark carbon fixation in this sponge species. Orthologues of *amoABC/pmoABC* were also found in Desulfobacterota from *P. ficiformis* and are attributed to methane oxidation as explained below. The *pmoA* subunit of this MAG was expressed in 37 out of the 39 *P. ficiformis* samples suggesting a wide distribution for methane oxidation in *P. ficiformis* (Fig. S7A).

### Carbon fixation measurements in sponges

Physiology experiments, using ^14^C-labeled bicarbonate can test the ability of autotrophic carbon assimilation that is light dependent (photosynthetic activity) or that occurs in darkness (dark primary production). Two sponge species were used in the ^14^C-labeled bicarbonate fixation experiments: (1) *P. ficiformis*, harboring *Ca*. S. feldmannii, and (2) *T. swinhoei*, with *Ca*. S. spongiarum. The latter sponge is also known to harbor a dense population of filamentous Tectomicrobia that have genomic potential to fix carbon *via* CBB, utilizing multiple inorganic energy sources (Table S5, Fig. 1).

**Fig. 6:**
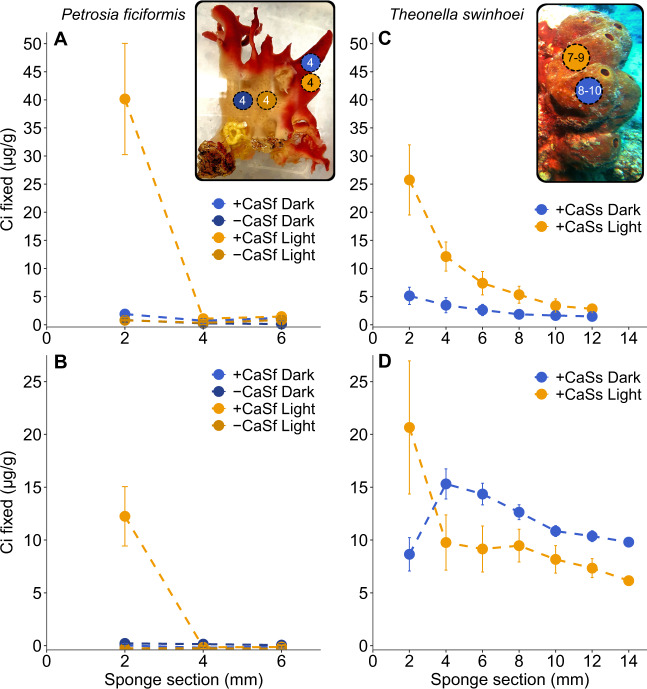
Light and dark carbon fixation in *P. ficiformis* and *T. swinhoei* sponge tissue measured using NaH^14^CO_3_ radioisotope assays. The *y* axis represents amounts (µg/g) of fixed Ci across parallel sections of the sponge (x axis), including the most external (outer 2 mm, harboring cyanobacterial symbionts) and internal (Cyanobacteria-free) sections. Image inserts—pictures of *P. ficiformis* (left) and *T. swinhoei* (right), circles schematically represent the number of cylinders that were cut in each experiment. **A**, **B** Two experiments conducted on *P. ficiformis* precut cylinders. Cylinders derived from pink (Cyanobacteria harboring) and white (Cyanobacteria-free) sponge surfaces. 16 cylinders (including 12 live and the 4 kill controls) were cut from each *P. ficiformis* specimen. One specimen of *P. ficiformis* was used in each experiment (two sponge specimens in total). Cylinders were incubated with NaH^14^CO_3_-labeled seawater in light and dark for 2 h and then cut into three sections to establish the amount of fixed carbon in the outer and inner sponge layers. Mean ± SD (*n* = 3 cylinders for light and *n* = 3 cylinders for dark conditions, “n” is the number of live cylinders for each condition and does not include the cylinders that were used as kill controls). **C**, **D** *T. swinhoei*, two specimens (one for light and one for darkness exposures, respectively) were used for each of two experiments performed (four sponge specimens in total). Sponge specimens were incubated in NaH^14^CO_3_-labeled seawater in light or in darkness for 2 h. At the end of the incubation, 7–10 cylinders were cut out of each *T. swinhoei* specimen and each cylinder was divided into 6–7 sections to establish the amount of fixed carbon in the cortex and in the inner sponge layers. Mean ± SD (*n* = 9 (**C**) and *n* = 7 (**D**) for light, and *n* = 10 (**C**) and *n* = 8 (**D**) cylinders for dark conditions). CaSs *Ca*. S. spongiarum. CaSf *Ca*. S. feldmannii.

Light-mediated inorganic carbon fixation was detected in both species in the cortex (external layer) of the sponge, where Cyanobacteria reside. While on average *ca*. 81–97% of total (i.e., light+dark) carbon fixation occurred in light conditions in *P. ficiformis* (Fig. 6A, B), the overall contribution of light fixation in *T. swinhoei* ranged between 46 and 78% (Fig. 6C, D). We associated the observed decreasing gradient of the labeled fixed carbon across inner sponge sections (≥4 mm) with a transfer of photosynthates to internal sponge layers. This transfer was only observed for *T. swinhoei* (Fig. 6C, D). In contrast, the labeled photosynthates produced by *Ca*. S. feldmannii remained within the cortex of *P. ficiformis* (Fig. 6A, B). The lack of contribution of fixed organic carbon from *Ca*. S. feldmannii to internal layers of the sponge host supports the previous hypothesis that the symbiotic role of this photosymbiont may not be directly related to its photosynthetic properties, and rather to protection from solar radiation via synthesis of pigments [32]. Accordingly, presence of a photosymbiont does not directly imply transfer of organic carbon to its host, and alternative benefits need to be investigated. Diverse trends in carbon contribution to the host were shown also for different sponge species harboring *Ca*. S. spongiarum, and it was suggested that such variability may relate to symbiont phylotypes (clades within *Ca*. S. spongiarum) [12, 27].

Regarding chemosynthetic or dark carbon fixation, Nitrospirota and Thaumarchaeota symbionts were reported in both *T. swinhoei* and *P. ficiformis*, phyla that we show here are capable of dark carbon fixation via the rTCA and 3-HP/4-HB cycles, respectively. Both cycles are energetically fueled by different stages of nitrification, with ammonia and nitrite oxidation processes driven by Thaumarchaeota and Nitrospirota, respectively. However, ammonia oxidation rates were shown to be ten times lower than nitrite oxidation rates in the Mediterranean sponges *Dysidea avara* and *Chondrosia reniformis* [95], suggesting a larger influence of the rTCA compared to the 3-HP/4-HB cycle in dark carbon fixation. If a similar trend is relevant to *P. ficiformis*, then Thaumarchaeota may contribute little fixed carbon, resulting in the very low dark fixation observed in the ^14^C-labeling experiments. Nitrospirota were reported to be present at low relative abundance and have low transcriptional activity in *P. ficiformis* [32], which may have resulted in the low impact of species of this phylum on the dark carbon fixation measured for this sponge. A different trend was reported for *T. swinhoei* (South China Sea) where the abundance of genes and transcripts related to ammonia oxidation was higher than those related to nitrite oxidation [96]. Accordingly, *T. swinhoei* may have higher rates of the dark carbon fixation via the 3-HP/4-HB and rTCA cycles compared to *P. ficiformis*.

Chemosynthetic or dark carbon fixation in *T. swinhoei* represented 16.6–29.5% of total fixation. In addition to symbiotic Thaumarchaeota [35] and Nitrospirota [97]*, T. swinhoei* is also known to harbor abundant filamentous Tectomicrobia (“Entotheonella”). This symbiont as well as Thaumarchaeota and Nitrospirota may be responsible for the observed dark fixation in this sponge species. Tectomicrobia may use the CBB cycle fueled by a wide range of inorganic energy sources. In contrast to *T. swinhoei*, dark fixation contributed very little to total fixation (0.1–4.5%) in *P. ficiformis*. Italian *P. ficiformis* specimens harbor Gammaproteobacteria symbionts that are capable of non-photosynthetic CBB fixation, as shown in this study based on the detection of gammaproteobacterial *rbcL* transcripts. However, this chemosynthesis may not be relevant for Israeli *P. ficiformis* specimens, where homologues of the same *rbcL* gene were not detected in metagenomes or MAGs (Figs. 1,  S8, Supplementary File S1, Table S6). Accordingly, and supported also by the ^14^C-label experiments conducted on Israeli *P. ficiformis* specimens, chemoautotrophic pathways have only a minor influence on the overall carbon fixation compared to the photoautotrophic activity of *Ca*. S. feldmannii.

A decreasing pattern in H^14^CO_3_^−^ concentration in the medium in which the *P. ficiformis* cylinders were incubated was observed for both the Cyanobacteria-harboring cylinders (where H^14^CO_3_^−^ was fixed by *Ca*. S. feldmannii) (Fig. S9A, B), and the white cylinders without Cyanobacteria (Fig. S9A, C). Killed samples (formalin controls) did not show decreasing patterns of H^14^CO_3_^−^ in the incubation medium (Fig. S9), implying biologically active uptake in living cylinders in the dark as well as the light. Given the minimal dark fixation observed, we speculate that the uptake of H^14^CO_3_^−^ by the sponge in the dark resulted from assimilation of bicarbonate via anaplerotic reactions followed by immediate respiration of most of the assimilated carbon to CO_2_.

If the dark-fixed H^14^CO_3_^−^ was indeed immediately respired back to CO_2_, we should only have detected the decrease in labeled H^14^CO_3_^−^ in the seawater if it had remained trapped inside the sponge tissue. We thus conducted an additional experiment with white (Cyanobacteria-free) *P. ficiformis* cylinders incubated with H^14^CO_3_^−^ in the dark, and once the decrease of labeled H^14^CO_3_^−^ in the medium was detected, we crushed the sponge tissue. This resulted in an increase of label in the medium indicating release of the labeled CO_2_ from the sponge cylinders back to the medium (Fig. S9C). This supports a fast turnover of dark-fixed CO_2_ in *P. ficiformis*, which might be related to anaplerotic carbon assimilation. Further, our results suggest that anaplerotic carbon assimilation in *P. ficiformis* likely results in energy production rather than in biomass accumulation.

The anaplerotic rates in the laboratory conditions may be lower than in the natural environment due to differences in accessibility to metabolically important compounds [98] such as electron donors (e.g., pelagic CO). In fact, physiological experiments performed on planktonic Gammaproteobacteria showed increased rates of anaplerotic Ci assimilation when the appropriate energy source (e.g., thiosulfate) and anaplerotic carbon acceptor (e.g., pyruvate) were added [20]. We therefore cannot exclude the possibility that anaplerotic carbon assimilation in laboratory conditions might be different from the natural conditions.

## Conclusions

Most sponge symbionts were found to be lithoheterotrophs or organoheterotrophs with the exception of taxonomically restricted groups of autotrophs that implement the 3-HP/4-HB, CBB, and rTCA pathways. Anaerobic forms of CODH and the WL pathway, previously suggested as being part of the Ci-fixing metabolic repertoire of some sponge symbionts, were found to be absent from the sponge microbiome. CO oxidation driven by the aerobic form of CODH was found to be ubiquitous in sponge symbionts, likely representing the main inorganic energy source for lithoheterotrophs. Different variations of CODH and *amoABC*/*pmoABC* found across symbiotic lineages have evolved towards oxidation of diverse inorganic (e.g., CO and ammonia) and organic (e.g., xanthine and methane) compounds. The sources of these compounds may be ambient seawater that is continuously pumped through the sponge water channels or holobiont metabolism. Certain symbionts might use these energy sources for chemosynthetic carbon fixation. Our experiments provide evidence for dark fixation in sponges, in particular for *T. swinhoei*. Dark fixation processes in *P. ficiformis* (and potentially other sponge species) may also involve anaplerotic carbon assimilation, which is likely carried out by Acidobacteria and possibly also by Alphaproteobacteria and Chloroflexi. Finally, we showed that cyanobacterial *Parasynechococcus-*like symbionts are highly diverse in terms of their contributions to the overall holobiont carbon budget, with *Ca*. S. spongiarum sharing its photosynthates with the host and *Ca*. S. feldmannii behaving as a “selfish” guest.

## Supplementary information

Supplementary File S1 (text) and supplementary figures (S1-S9)

File_S2

File_S3

Supplementary tables (S1-S6)

## Data Availability

MAGs from this study can be found under NCBI bioprojects ID PRJNA515489 (*P. ficiformis*), PRJNA255756 (*T. swinhoei*), PRJNA712987 (*A. aerophoba*), and PRJNA273429 (*I. variabilis*).
