# Correction: Lineage-specific energy and carbon metabolism of sponge symbionts and contributions to the host carbon pool

**DOI:** 10.1038/s41396-022-01186-y

**Published:** 2022-01-19

**Authors:** I. Burgsdorf, S. Sizikov, V. Squatrito, M. Britstein, B. M. Slaby, C. Cerrano, K. M. Handley, L. Steindler

**Affiliations:** 1 Department of Marine Biology, Leon H. Charney School of Marine Sciences, University of Haifa, Haifa, Israel; 2 GEOMAR Helmholtz Centre for Ocean Research Kiel, RD3 Marine Ecology, RU Marine Symbioses, Kiel, Germany; 3 Department of Life and Environmental Sciences, Polytechnic University of Marche, Ancona, Italy; 4 School of Biological Sciences, The University of Auckland, Auckland, New Zealand

**Keywords:** Metabolism, Functional genomics, Bacterial genetics, Comparative genomics, Water microbiology

Correction to: *The* *ISME Journal* 10.1038/s41396-021-01165-9

The article “Lineage-specific energy and carbon metabolism of sponge symbionts and contributions to the host carbon pool”, written by I. Burgsdorf, S. Sizikov, V. Squatrito, M. Britstein, B. M. Slaby, C. Cerrano, K. M. Handley, L. Steindler, was originally published electronically on the publisher’s internet portal on 7 December 2021 without open access. With the author(s)’ decision to opt for Open Choice the copyright of the article changed on 29 December 2021 to © The Author(s) 2021 and the article is forthwith distributed under a Creative Commons Attribution 4.0 International License, which permits use, sharing, adaptation, distribution and reproduction in any medium or format, as long as you give appropriate credit to the original author(s) and the source, provide a link to the Creative Commons licence, and indicate if changes were made. The images or other third party material in this article are included in the article’s Creative Commons licence, unless indicated otherwise in a credit line to the material. If material is not included in the article’s Creative Commons licence and your intended use is not permitted by statutory regulation or exceeds the permitted use, you will need to obtain permission directly from the copyright holder. To view a copy of this licence, visit http://creativecommons.org/licenses/by/4.0/.

